# Building an Adverse Outcome Pathway network for COVID-19

**DOI:** 10.3389/fsysb.2024.1384481

**Published:** 2024-06-06

**Authors:** Penny Nymark, Laure-Alix Clerbaux, Maria-João Amorim, Christos Andronis, Francesca de Bernardi, Gillina F. G. Bezemer, Sandra Coecke, Felicity N. E. Gavins, Daniel Jacobson, Eftychia Lekka, Luigi Margiotta-Casaluci, Marvin Martens, Sally A. Mayasich, Holly M. Mortensen, Young Jun Kim, Magdalini Sachana, Shihori Tanabe, Vassilis Virvilis, Stephen W. Edwards, Sabina Halappanavar

**Affiliations:** 1 Institute of Environmental Medicine, Karolinska Institute, Stockholm, Sweden; 2 Institute of Clinical and Experimental Research (IREC), UCLouvain, Brussels, Belgium; 3 European Commission, Joint Research Centre (JRC), Ispra, Italy; 4 Research Centre, Universidade Católica Portuguesa, Lisboa, Portugal; 5 Instituto Gulbenkian de Ciência, Fundação Calouste Gulbenkian, Oeiras, Portugal; 6 Biovista, Athens, Greece; 7 Division of Otorhinolaryngology, Department of Biotechnologies and Life Sciences, University of Insubria, Ospedale di Circolo e Fondazione Macchi, Varese, Italy; 8 Impact Station, Hilversum, Netherlands; 9 Department of Pharmaceutical Sciences, Utrecht University, Utrecht, Netherlands; 10 The Centre for Inflammation Research and Translational Medicine, Brunel University London, London, United Kingdom; 11 Biosciences, Oak Ridge National Laboratory, Oak Ridge, TN, United States; 12 King’s College London, London, United Kingdom; 13 Department of Bioinformatics (BiGCaT), NUTRIM, Maastricht University, Maastricht, Netherlands; 14 Aquatic Sciences Center, University of Wisconsin-Madison at US EPA, Duluth, MN, United States; 15 U.S. Environmental Protection Agency (US EPA), Research Triangle Park, NC, United States; 16 Environmental Safety Group, KIST Europe, Saarbrucken, Germany; 17 Environment Health and Safety Division, Environment Directorate, Organisation for Economic Cooperation and Development (OECD), Paris, France; 18 National Institute of Health Sciences, Kawasaki, Japan; 19 RTI International, Research Triangle Park, NC, United States; 20 Environmental Health Science and Research Bureau, Health Canada, Ottawa, ON, Canada; 21 Department of Biology, University of Ottawa, Ottawa, ON, Canada

**Keywords:** COVID-19, adverse outcome pathway (AOP), network, inflammation, event

## Abstract

The COVID-19 pandemic generated large amounts of data on the disease pathogenesis leading to a need for organizing the vast knowledge in a succinct manner. Between April 2020 and February 2023, the CIAO consortium exploited the Adverse Outcome Pathway (AOP) framework to comprehensively gather and systematically organize published scientific literature on COVID-19 pathology. The project considered 24 pathways relevant for COVID-19 by identifying essential key events (KEs) leading to 19 adverse outcomes observed in patients. While an individual AOP defines causally linked perturbed KEs towards an outcome, building an AOP network visually reflect the interrelatedness of the various pathways and outcomes. In this study, 17 of those COVID-19 AOPs were selected based on quality criteria to computationally derive an AOP network. This primary network highlighted the need to consider tissue specificity and helped to identify missing or redundant elements which were then manually implemented in the final network. Such a network enabled visualization of the complex interactions of the KEs leading to the various outcomes of the multifaceted COVID-19 and confirmed the central role of the inflammatory response in the disease. In addition, this study disclosed the importance of terminology harmonization and of tissue/organ specificity for network building. Furthermore the unequal completeness and quality of information contained in the AOPs highlighted the need for tighter implementation of the FAIR principles to improve AOP findability, accessibility, interoperability and re-usability. Finally, the study underlined that describing KEs specific to SARS-CoV-2 replication and discriminating physiological from pathological inflammation is necessary but requires adaptations to the framework. Hence, based on the challenges encountered, we proposed recommendations relevant for ongoing and future AOP-aligned consortia aiming to build computationally biologically meaningful AOP networks in the context of, but not limited to, viral diseases.

## 1 Introduction

SARS-CoV-2 infection provokes the coronavirus disease 2019 (COVID-19). Patients can experience a range of clinical manifestations, from no symptoms to critical illness and death. Underlying mechanisms of COVID-19 have been extensively investigated and were instrumental for the implementation of rapid therapeutic and preventive measures. However, this unprecedented worldwide investigation of a disease resulted in an overwhelming flux of *in vitro*, *in vivo*, clinical and epidemiological data regarding COVID-19 pathogenesis. In this context, the CIAO project (https://www.ciao-covid.net/) was initiated early 2020 by the Joint Research Centre of the European Commission as a crowdsourcing initiative comprising more than 70 scientists from academic, regulatory and industrial organizations worldwide (Nymark et al., 2021). The aim of the project was to comprehensively gather and systematically organize published scientific literature on COVID-19 pathology using the Adverse Outcome Pathway (AOP) framework.

An AOP describes a series of biological Key Events (KEs) that are essential for the initiation and progression of the disease, of which the Molecular Initiating Event (MIE) is the first one (Villeneuve et al., 2014; OECD, 2018). MIE describes the molecular interaction between a stressor and the biological system, responsible for initiating the downstream cascade of KEs, culminating in the manifestation of an Adverse Outcome (AO). The Key Event Relationships (KERs) describe the causal relationship between an upstream and a downstream KE. In essence, the series of selected set of molecular, cellular and tissue level KEs and KERs presented in an AOP are expected to predict the occurrence of an AO at the tissue, organism, individual or population level.

Within the CIAO project, 22 AOPs were partially or fully developed spanning multiple organs such as respiratory systems, nervous system, gastrointestinal tract and liver (Wittwehr et al., 2021; Clerbaux, Amigó, et al., 2022). Around 70 KEs were identified occurring at different levels of biological organization that play a role in initiation, promotion, and clinical manifestation of COVID-19. Around half of these KEs were already described in the online repository platform AOP-Wiki (www.aop-wiki.org), such as the set of harmonized KEs representing inflammation (Villeneuve et al., 2019) re-used to build the inflammatory response of the disease. The remaining >30 KEs were identified and added to the AOP-Wiki during the CIAO project.

Fourteen of those AOPs start with the same MIE, namely, “binding to angiotensin converting enzyme-2 (ACE2)” receptor. For 7 of those, binding to ACE2 leads to viral replication as the virus mainly uses the ACE2 receptor to enter the cell and replicate. Viral replication is essential to drive some outcomes, hence it was proposed to develop a *viral hub AOP* consisting of SARS-CoV-2 specific KEs depicting its replication steps (Clerbaux et al., 2023).

Regarding AOs, the 22 CIAO AOPs depict pathways leading to 19 outcomes of the multifaceted COVID-19, namely, infection proliferation, thrombosis, microvascular dysfunction, hyperinflammation, thrombo-inflammation, acute respiratory distress, lung fibrosis (x2), decreased lung function, encephalitis, short-term anosmia, stroke, dysgeusia, intestinal barrier disruption, gut dysbiosis, liver fibrosis, liver injury (x2), kidney injury (x2), heart failure and myocardial infraction. The evidence supporting the development of the pathways leading to short-term anosmia, intestinal disorders and gut dysbiosis have been described in detail by the consortium (Clerbaux, Fillipovska, et al., 2022; Clerbaux, Mayasich, et al., 2022; Shahbaz et al., 2022). AOPs related to neurological symptoms in COVID-19 such as stroke, encephalitis and others have been detailed in Hogberg et al., 2022. The 2 AOPs leading to liver injury propose an indirect effect via systemic inflammation or due to hypoxia/thrombosis following binding to ACE2 in the lungs (Vinken, 2021). The 2 AOPs leading to kidney injury were built on the similar rationale (Wittwehr et al., 2021). Those 4 liver and kidney related AOPs were not added into the AOP-Wiki.

In addition, lung fibrosis and decreased lung function were already reported in the AOP-Wiki to be triggered by certain chemicals and nanomaterials (Gerloff et al., 2017; Da et al., 2021). Similarities in responses to SARS-CoV-2 and those nanomaterials or chemicals suggest common pathways leading to lung fibrosis and decrease lung function highlighting mechanistic cross-talk between (nano) toxicology and viral diseases (Gerloff et al., 2017; Kim et al., 2021; Kinaret, del Giudice, and Dario, 2020). Those AOPs were further considered in the project (Clerbaux, Amigó, et al., 2022), resulting in a total of 24 individual linear AOPs linked to COVID-19. From those 24 AOPs, 20 are present in the AOP-Wiki and 11 were included under the CIAO 1.96 Organisation for Economic Co-operation and Development (OECD) AOP program work plan but have, however, not yet completed the OECD reviewing process at this date.

Individual AOPs are pragmatic units describing how defined perturbations of a biological system can lead in a causal manner to a particular AO. However, to reflect the complex biology and interrelatedness of the various processes, a network of AOPs can be generated that can, in turn, guide AOP development (Knapen et al., 2018; Villeneuve et al., 2018). In an AOP network derivation, shared KEs or hub node revealed after AOPs have been developed independently (Knapen et al., 2018).

The objective of this study was to derive a directed network of AOPs for COVID-19. The primary network was used to inform the curation procedure highlighting the inconsistencies in AOP nomenclature and harmonization opportunities. After manual refinement, the final network enabled visualization of the complex interactions of the KEs leading to the various AOs of COVID-19. Based on the challenges encountered in deriving and visualizing a biologically relevant Wiki-derived AOP network for COVID-19 and building further on previous efforts (Wiklund et al., 2023), we propose here recommendations to address issues such as ontology standardization and organ specificity in the AOP-Wiki. We also discuss ways to tackle quality issues in individual AOPs in the AOP-Wiki such as unequal completeness in KE description and limited scope of available evidence provided in KERs. These quality issues are acknowledged as reflecting the need for further implementation of the FAIR principles to improve AOP findability, accessibility, interoperability and re-usability (Wittwehr et al., 2024). Finally, the study highlights the specific challenges of exploiting AOPs for a viral disease such as the need for capturing KEs specific to SARS-CoV-2 replication while complying with the stressor agnostic principle of the framework. Thus, this study proposes recommendations linked to the challenges encountered during development of an AOP network for modeling diseases as well as for assessing toxicity.

## 2 Methods & materials

### 2.1 Manual extraction and inclusion of relevant AOPs

AOPs describing KEs involved in COVID-19 were collected manually. This included the individual AOPs developed within the CIAO project and AOPs already available in the AOP-Wiki. The AOPs that did not have a unique AOP-Wiki identifiers (IDs), that were not compliant with the OECD AOP development guidance (OECD, 2018), that lacked KER components and that were incomplete with only KEs or KERs titles in the AOP-Wiki, were excluded from the analysis. All AOPs and associated KEs/KERs included in the network are available in the AOP-Wiki and publicly accessible under a CC BY 4.0 license.

### 2.2 Systematic and manual pre-processing of the KE and KERs contained in the AOPs

The KEs and KERs contained in the selected AOPs were retranscribed in a spreadsheet (Supplementary Material S1, first sheet). In addition, an expert analysis was also performed to identify KE that describe the same biological perturbation but under different KE titles and manually merge them under a single ID while retaining their original AOP-Wiki IDs in the spreadsheet (Supplementary Material S1, second sheet). To account for KE duplications frequently observed in AOP-Wiki, a systematic way to map KE titles to standard ontology terms derived from various ontologies was used (Supplementary Material S1 1, third sheet). This is a crucial step towards establishing an interoperability across different AOPs, needed for computational generation of a shared node in the network.

### 2.3 Computational derivation of a primary network

The pre-processed information (S1) was used to generate a directional AOP network using the open-source network visualization program Cytoscape v3.7 through a series of iterative processes. The spreadsheet (S1) was imported through Cytoscape “import network from file” function. Interaction parameters and attributes were defined considering directionality of KEs in the AOPs, including source and target nodes in order to allow the program to identify KERs (i.e., connectivity between downstream to upstream KEs), interaction type (adjacent or nonadjacent), and edge, target and source node attributes (AOP and KE type, e.g., whether a node was a MIE, KE or an AO). The attributes were used to define visualization options, such as colored nodes with the MIE in green, KEs in yellow/orange and the AO in red. The edge (i.e., KER) colors correspond to individual AOPs. The Cytoscape app NetworkAnalyzer was applied to analyze the directed network properties.

The primary network could also be derived from the third party tool Biovista Vizit based on the selected AOPs and expansion of the KEs with a minimum effort on node rearrangement.

### 2.4 Manual refinement of the primary network

The computationally generated primary network was further refined and organized in a tissue specific manner. This required duplication of some MIEs and KEs as well as integration of missing elements.

## 3 Results

### 3.1 Manual extraction of the relevant AOPs

As part of the CIAO project, 22 AOPs have been developed of which 18 were added into the AOP-Wiki and 11 were included under the 1.96 OECD AOP program work plan (Table 1). Binding to ACE2 is the MIE of 14 of those AOPs. Binding to ACE2 leads to viral replication for 7 of them (AOP320, 379, 394, 395, 422, 428 and 430) as the virus mainly uses this receptor to enter the cell and replicate. While for 4 AOPs (AOP381, 383, 385, 428), binding to ACE2 induces ACE2 dysregulation/downregulation depicting the effect on the physiological role in the renin-angiotensin system (RAS) of ACE2 when hijacked by SARS-CoV-2.

In addition to the AOPs developed within the CIAO project, 2 AOPs already present in the AOP-Wiki, namely, AOP173 developed under OECD project 1.32 and AOP302 developed under OECD project 1.87, described pathways that were relevant for COVID-19 (Da et al., 2021; Halappanavar et al., 2023). Taking advantage of the stressor agnostic principle of the framework which bridge knowledge of mechanisms of adversity, those 2 AOPs were further considered for COVID-19.

In total, 24 AOPs were collected, of which 17 were considered for inclusion for the primary network derivation and analysis. The other AOPs were excluded as they were not present in the Wiki or not following the OECD guidance (Table 1).

### 3.2 Systematic and manual pre-processing of the information contained in the AOPs

Systematic analysis of KEs containing similar KE titles, resulted in the identification of the following KEs as potential duplicates: KE1276 “Lung fibrosis” and KE1458 “Pulmonary fibrosis”. An additional expert judgment was necessary to manually merge 16 additional paired KEs that are similar with regards to the biological perturbations that they describe. A common title was arbitrary given for the purposes of network analysis (Table 2).

### 3.3 Computational derivation of a primary network

**FIGURE 1:**
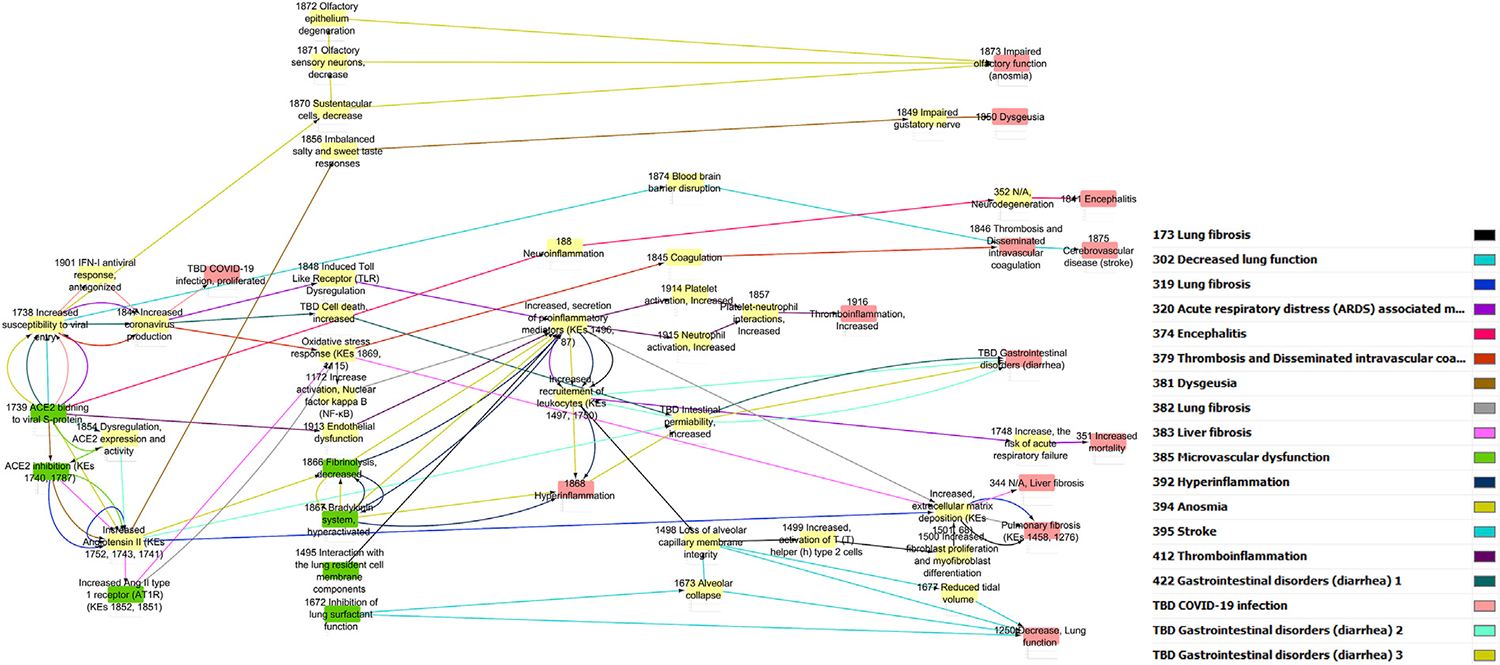
Computational derivation of a primary network using Cytoscape.

The pre-processed information was used to generate a directional AOP network using Cytoscape through a series of iterative processes (Figure 1). Nodes were colored in green for MIE, yellow for KEs and red for AO. The edge (i.e., KER) colors differentiate individual AOPs.

**FIGURE 2:**
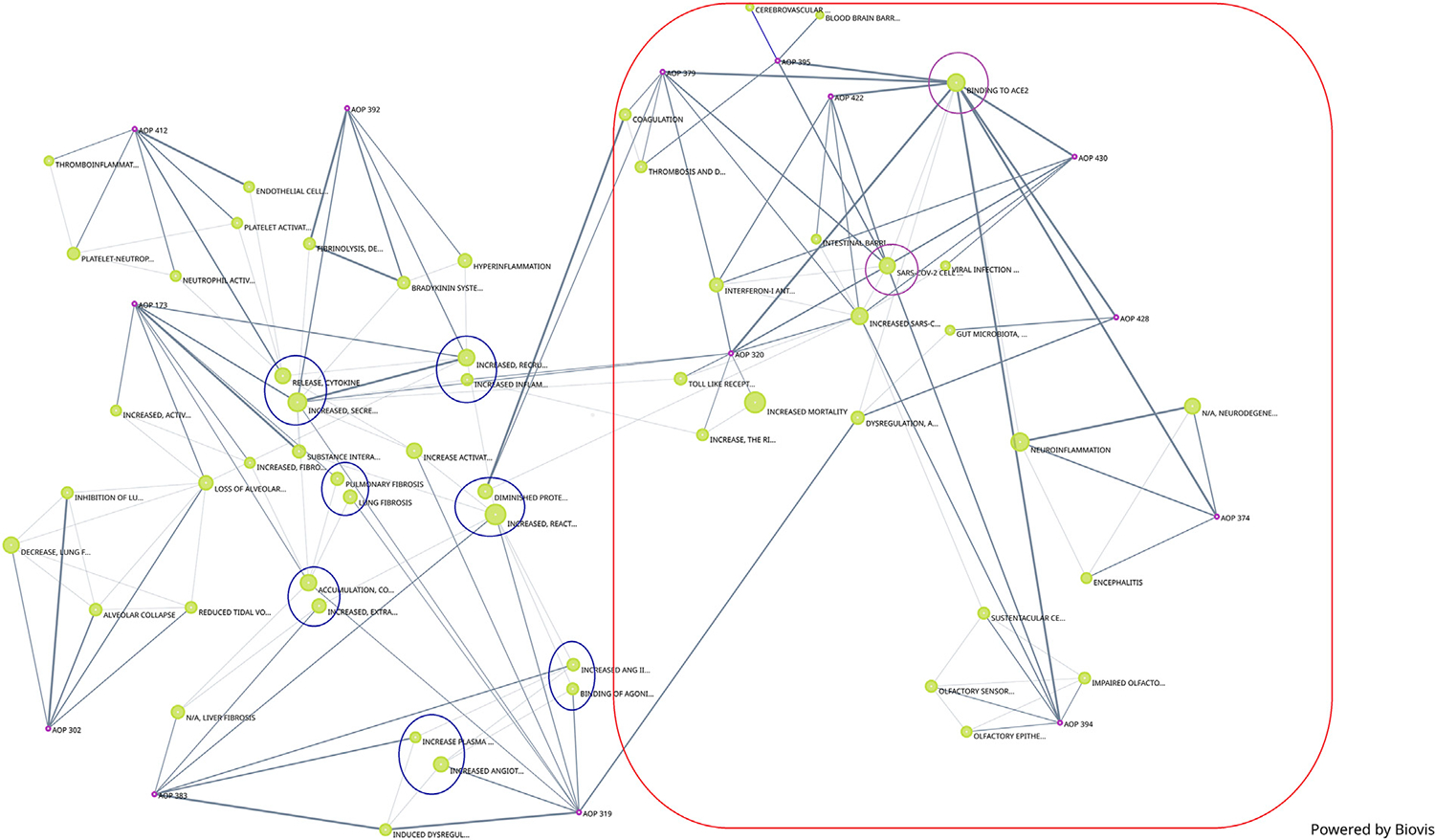
Network of COVID-19 AOPs as generated by Biovista Vizit tool.

In addition to the network generated by Cytoscape, a network generated by Biovista Vizit as a live editable graph (Figure 2) is provided. For clarity, circles were drawn around the harmonized KEs (blue circles in Figure 2).

The use of an automated tool for visualization makes the high-level inspection of the graph easier. The Vizit graph can be divided into two groups of AOPs: the ones that have ACE2 Binding KE-and associated downstream and upstream KEs (purple circles and red box) and others that do not. At the top of the graph, 2 KEs are not connected; KE1874 “Blood brain barrier disruption” and KE1875 “Cerebrovascular disease (stroke)”, both of which are part of AOP395 “Binding of Sars-CoV-2 spike protein to ACE2 receptors expressed on pericytes leads to stroke”.

### 3.4 Manual refinement of primary network

#### 3.4.1 Identification of missing KEs and emergence of a new AOP

The primary network highlighted the fact that cell death, widely acknowledged as an essential and crucial step in disease pathogenesis (Deshpande and Zou, 2021; Li et al., 2022; Tojo, 2023; Yuan et al., 2023) was not present in any of the AOPs, hence in the network. Therefore, the KE “cell death” KE1825 present in the Wiki was added as a new node connecting “increased coronavirus production” (KE1847 from AOP430) to “increased, secretion of proinflammatory mediators (KE 1496 from AOP392). With this, a new AOP emerged: “Binding of SARS-CoV-2 to ACE2 leads to hyperinflammation via cell death” which was added to the AOP-Wiki (AOP468).

#### 3.4.2 Merging of two identical AOPs

The primary network also highlighted that AOP382 and AOP319 described overlapping KEs of a same pathway involving RAS up to lung fibrosis and have been therefore merged into one AOP (AOP319) as such: ACE2 downregulation > Ang II accumulation > Ang II-AT1R activation > ROS regeneration > Collagen deposition > lung fibrosis.

#### 3.4.3 Duplication of tissue specific MIEs and KEs

During manual refinement of the primary network, a need for visualizing tissue-specificity for the MIEs and early KEs was identified.

First, the MIE describing “binding to ACE2” (MIE1739) and the early viral KEs, namely, KE1738 (SARS-CoV-2 cell entry) and KE 1847 (Increased SARS-CoV-2 production) were replicated. Replication of the KEs (*i.e*., nodes in the network) was accompanied by creation of a new tissue specific KER annotated with the relevant AOP-belonging of the KE. This step was important to distinguish between the entry routes. For example, viral entry through sustentacular cells (nasal), through pulmonary cells (lungs), or through enterocytes (gut) had to be differentiated, as the downstream KEs and AOs will be different depending on the organ. Not duplicating tissue specific MIEs would have led to a network that is not biologically meaningful. For example, without that manual refinement, binding to ACE2 in enterocytes would lead to anosmia. In addition, a new previously undefined MIE, referring to ‘secondary’ routes of viral entry, was added to reflect potential proliferation of the virus to other organs via the systemic circulation (Kirtipal et al., 2022).

Second, the KE 1854 (Dysregulation of ACE2 expression and activity) was replicated to differentiate the effect of ACE2 dysregulation in enterocytes *versus* in lungs for the same rationale as described above.

#### 3.4.4 Network-guided identification of missing KERs

The addition of ‘increased cell death’ and “secondary entry routes” resulted in identification of 4 new KERs (one to four in Table 3). Besides, to capture the clinical outcomes in liver and kidney, AO344 “liver injury” and AO759 “increased kidney failure” were added as downstream KEs of thrombosis and hyperinflammation (Vinken, 2021) leading to the creation of 4 extra KERs (five to eight in Table 3). Based on additional mechanisms proposed in CIAO publications (Clerbaux, Mayasich, et al., 2022; Hogberg et al., 2022), 3 extra KERs were added linked to neurological syndromes and 7 related to gastrointestinal symptoms (9–18 in Table 3).

All those 18 KERs were added to the network as new directed edges connecting the nodes representing the upstream and downstream KEs. The majority of these KERs are however currently empty in the AOP-Wiki. The recently described pragmatic “unit approach” for development of single KER could be applied for these KERs in the future (Svingen et al., 2021).

### 3.5 Final network

AOPs are living documents that can be constantly updated as new information becomes available. However, AOP381 and AOP383 were still empty at the time of final network and were then removed. The final COVID-19 AOP network included a total of 14 AOPs: the 13 highlighted in bold in Table 1 and the newly emerged from primary network refinement (AOP468).

#### 3.5.1 Visualization

**FIGURE 3:**
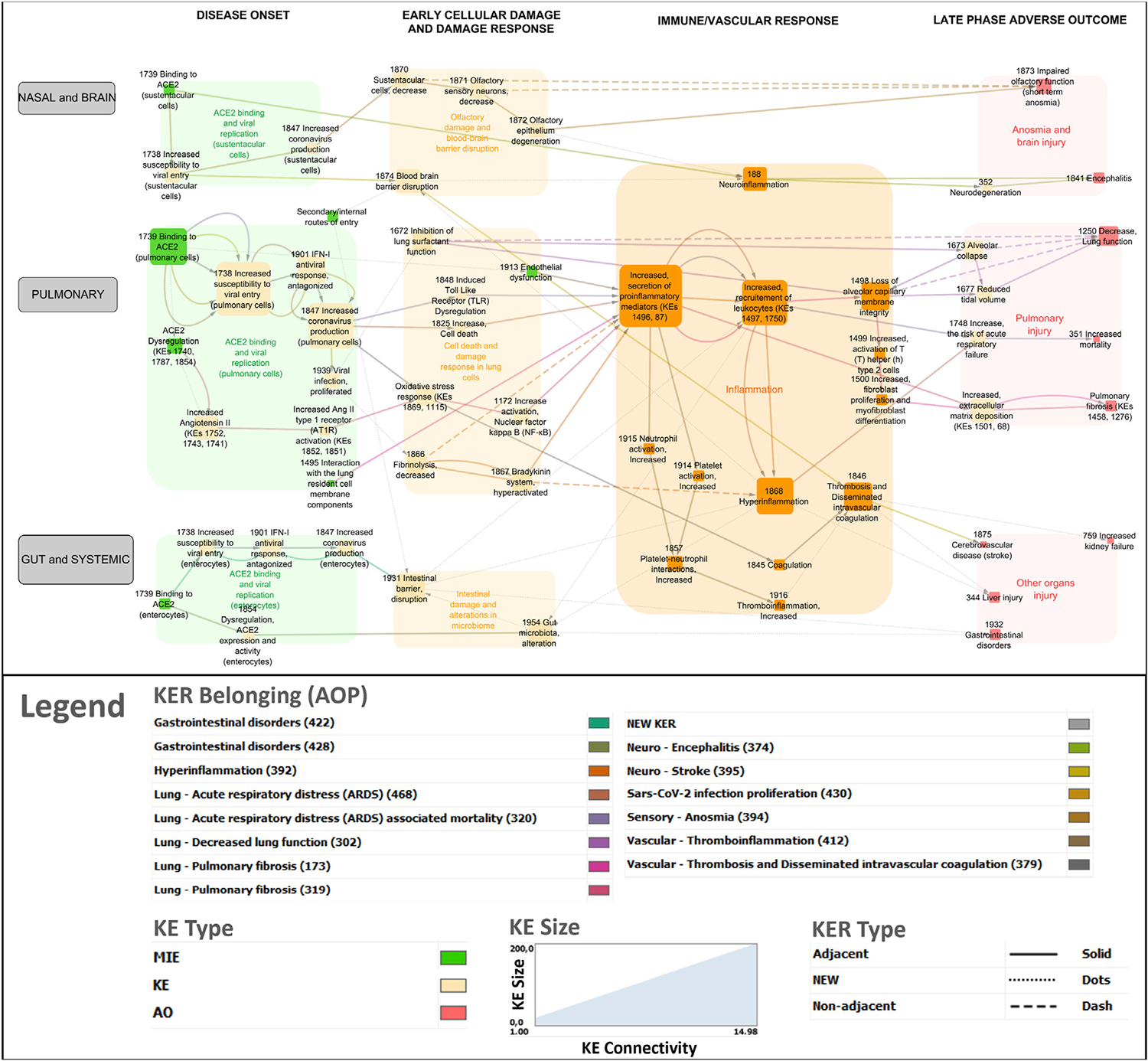
COVID-19 AOP network involving 14 individual adverse outcome pathways. Green nodes indicate molecular initiating events (MIEs), Orange nodes are key events (KEs) and Red nodes are adverse outcomes (AOs). Transparent boxes depict KEs including MIEs clustered by the disease phase and tissue specificity. The color code for KER belonging indicates which AOP a specific KER (edge in the network) belongs to.

The network was refined to distinguish between the different viral entry routes and consequent organ/system specific responses (nasal and brain, pulmonary, gut and systemic) (Figure 3). In addition, the network was divided into four distinct phases of disease progression involving biological events at the molecular, cellular and tissue levels, within each phase to address partially the challenge of the temporality of the disease not well-captured in AOPs.

- Disease onset phase - viral infection phase involving binding of SARS-CoV-2 to a cellular receptor, viral entry, antagonism of the antiviral response, replication and release of new virions as well as ACE2 dysregulation.
- Early cellular and tissue damage.
- Immune and vascular response phase - including the hyperinflammation response.
- Late adverse outcome phase: pulmonary failure, brain, gut and other organs injury.

#### 3.5.2 Topology

In total, 69 KEs formed the network with a clustering coefficient of 0.159. The average number of neighbors for each KE was 2.551, which indicates that each individual KE is connected (direct or indirect) with approximately two other KEs on average. The most highly connected KE was *Increased, secretion of proinflammatory mediators* (KE1496, KE87) with 14 connections creating a bow-tie topology to the network.

#### 3.5.3 Clustering

**FIGURE 4:**
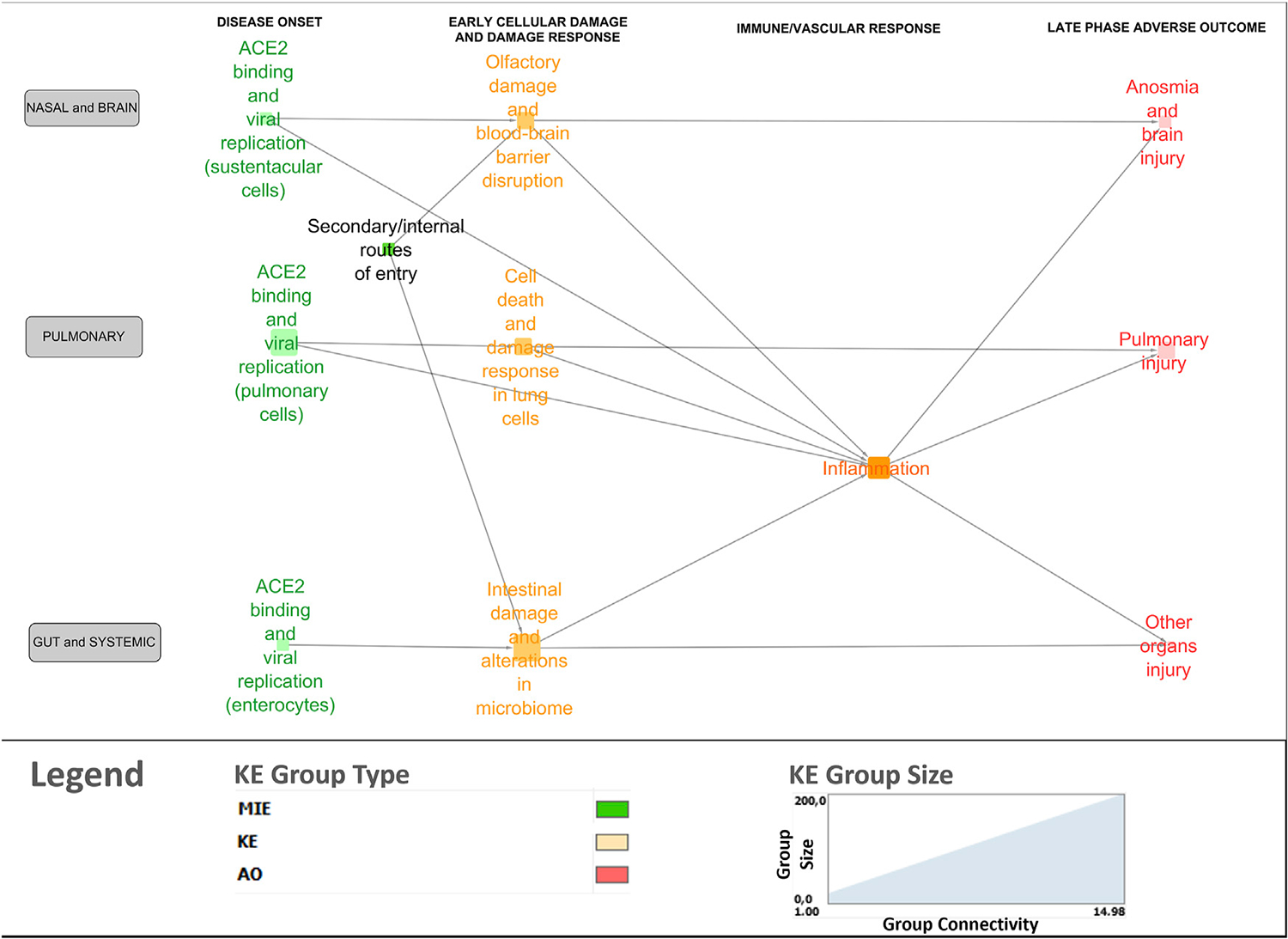
Bird’s-eye view of the COVID-19 AOP network providing insight into the interconnection between the various stages and tissue-specificities of the disease.

A bird’s-eye view version of the network was generated using the node clustering function in Cytoscape to cluster MIEs/KEs (nodes in the network) in line with overarching biological mechanisms and organ-specificity (Figure 4). Clustered nodes were collapsed by activating the expand/contract-function and visualizing it as a cluster including the descriptions of the core mechanisms. For example, the MIEs and KEs associated with the disease onset phase in the lungs were clustered and described under ACE2 binding and viral replication in pulmonary cells. The bird’s eye view supported a new level of insight in terms of expert-driven identification of gaps and discrepancies. For example, central KERs and KEs that had not been previously described in the 13 AOPs were identified, as described further below.

### 3.6 Brief description of the COVID-19 KEs and KERs

We aim here to briefly present the different KEs and KERs integrated in the network. The references and associated evidence used to support their essentiality in COVID-19 pathogenesis are documented in their AOP-Wiki IDs or are documented by references from the literature when KERs pages are empty.

#### 3.6.1 Disease onset phase

##### 3.6.1.1 Binding to receptor, viral entry, replication, and release of new virions

In the respiratory tract, SARS-CoV-2 spike proteins bind to the ACE2 receptor expressed at high levels on airway epithelial cells, alveolar epithelial cells, vascular endothelial cells and macrophages (KE1739, Binding to ACE2). Upon binding, the S protein subunits undergo sequential cleavage mediated by proteases and conformational changes that result in viral entry into the cells (KER2056). Many proteases, such as transmembrane serine protease 2 (TMPRSS2) have been identified to aid the virus with its cellular entry (KE1738, SARS-CoV-2 cell entry). Once inside of a cell, the virus undergoes replication (KER2496). The SARS-CoV-2 virus has evolved with a repertoire of proteins that bind and block proteins in the interferon (IFN) cascade primarily inhibiting the expression of host antiviral proteins, enabling replication of the virus (KE 1901, IFN-1 antiviral response, antagonized). When the antiviral response is antagonized, the viral RNA is translated, replicated, transcribed and the genomic RNA is packaged (KER2497) and the new SARS-CoV-2 virions are assembled (KE1847, Increased SARS-CoV-2 production). Subsequently, the newly formed virions are ready to infect an adjacent cell or another human via respiratory droplets, repeating the viral replication and infection cascade (KE 1939, Viral Infection, proliferated).

The virus can bind to any cell that expresses the ACE2 receptor on its surface along the path of the respiratory tract and, when distributed systemically, can bind to ACE2 positive cells in other organs (KE1739). ACE2 is also expressed on sustentacular cells of the olfactory neuroepithelium and enterocytes in the small intestine (KE1739). Hence, sustentacular cells could be infected as well as enterocytes via the olfactory and gastrointestinal routes respectively. And this has been represented in the network.

##### 3.6.1.2 ACE2 dysregulation

In addition to viral infection, the occupation of ACE2 receptors by the viral protein has been shown to dysregulate ACE2 expression and activity (KE1740, KE1787 and KE 1854, ACE2 Dysregulation). This leads to increased Angiotensin II (Ang II) (KE1752, KE1743 and KE1741, Increased, Angiotensin II) and increased expression and activity of its receptor Ang II type 1 (KE1852 - KE1851 Increased Ang II type I receptor (AT1R)), involved in RAS.

In the gastrointestinal tract, ACE2 also has a RAS-independent function in modulating dietary amino acid transport (KE 1854, (enterocytes)) and therefore, ACE2 dysregulation might interfere with intestinal homeostasis locally (Clerbaux et al.).

#### 3.6.2 Early cellular and tissue damage

##### 3.6.2.1 Cell death and damage response in pulmonary cells

In the lungs, SARS-CoV-2 is a cytopathic virus that causes extensive cell death (KE1825 Increase, cell death). KER linking SARS-CoV-2 replication to cell death has been created (KER2488).

Downregulation of ACE2 activity leads to activation of the tissue factor-dependent coagulation and inhibition of fibrinolysis (KE 1866, fibrinolysis, decreased) by Ang II (new KER in network). Internalization of ACE2 and hypofibrinolysis can lead to reduced degradation of bradykinin (KER2352), raising bradykinin levels and activities (KE 1867, Bradykinin system, hyperactivated), which could explain many of the symptoms associated with COVID-19, including vasodilation, hypotension, vascular permeability and hyperinflammation (KER2357).

Other KEs, such as KE1672 (Inhibition of lung surfactant function) and KE 1913 (Endothelial dysfunction), accentuate the damage and possibly set the stage for the severity of the ensuing pro-inflammatory response.

#### 3.6.3 Olfactory damage and blood-brain barrier disruption

In the olfactory epithelium, SARS-CoV-2 infection induces a decrease of sustentacular cell numbers (KE 1870, KER2545), which provide functional and structural support to the olfactory epithelium. The death of these supporting cells can lead to downregulation of olfactory sensory neurons (KE 1871, KER2362) and to degeneration in the structure and function of the olfactory epithelium (KE 1872, KER2363).

In addition to the nasal epithelium, there are other secondary routes of SARS-CoV-2 entry into the central nervous system (Hogberg et al., 2022). The invasion and infection of central nervous system lead to local immune response activation resulting in a cytokine storm, potentially provoking disruption of the blood brain barrier (BBB) (KE 1874, BBB disruption).

##### 3.6.3.1 Gastrointestinal damage

Under certain conditions, SARS-CoV-2 actively infects enterocytes potentially disrupting the intestinal barrier integrity (KE 1931, Intestinal barrier, disruption, KER2546), inducing a local inflammatory response in the gastrointestinal tract and fueling the systemic hyperinflammation (Clerbaux, Mayasich, et al., 2022). In addition, presence of SARS-CoV-2 in the gut has been shown to alter the gut microbiota (KE 1954, Gut microbiota alteration), potentially as a consequence of a disrupted intestinal barrier or because of the dysregulation of intestinal ACE2 (Clerbaux, Fillipovska, et al., 2022).

#### 3.6.4 Immune and vascular phase

Increased secretion of soluble factors including cytokines and chemokines (KE1496, Increased secretion of pro-inflammatory mediators) is the most common node in the network that is connected to several other KEs (Figure 1). Upstream KEs known to lead to release of pro-inflammatory mediators are dysregulated RAS, increased pro-inflammatory signaling (KE1172, Increase activation, Nuclear factor Kappa B (NFκB).

In the other direction, pro-inflammatory factors recruit immune cells (KER1703) including macrophages, monocytes and lymphocytes to the sites of infection (KE1497, KE1750, Increased recruitment of leukocytes). Immune cells further amplify the secretion of cytokines and chemokines, creating a pro-inflammatory environment (KER2761) leading to the pathological state of hyperinflammation (KE1866 Hyperinflammation) (KE2354). In addition, release of pro-inflammatory cytokines (KE1496) increases platelet activation (KE 1914) which increases neutrophil activation (KE915) increasing Platelet-neutrophil interactions (KE 1857) inducing Thromboinflammation (KE 1916). (KER2457 and KER2458).

During this phase, the endothelial injury leads to deregulation of thromboinflammatory processes, resulting in thrombus formation KE 1846 (Thrombosis and Disseminated intravascular coagulation), vascular injury and vascular dysfunction.

#### 3.6.5 Late adverse outcomes

##### 3.6.5.1 Pulmonary outcomes

Acute respiratory distress syndrome (ARDS, AO1748, Increased risk of respiratory failure) is one of the predominant AOs of the target organ in COVID-19. In survivors, Increased collagen deposition (KE68) leads to Pulmonary fibrosis (AO1458, AO1276). Hyperinflammation and lung tissue injury lead to Loss of alveolar capillary membrane integrity (KE1498), Reduced tidal volume (KE1677) and Alveolar collapse (KE1673) resulting in decreased lung function (AO1250).

##### 3.6.5.2 Brain injuries

Several neurological outcomes are commonly observed in COVID-19, including short-term anosmia (AO 1873), neurodegeneration (KE352), and encephalitis (AO 1841). Effects on the nervous systems could be the results of primary viral infection in the tissues or secondary to the target organ response in lungs (Kirtipal et al., 2022).

##### 3.6.5.3 Gastrointestinal disorders and multi-organ failure

Some COVID-19 patients also presented gastrointestinal symptoms such as diarrhea, abdominal discomfort, nausea and/or vomiting (AO 1932) and gut dysbiosis (KER2532).

As the disease spreads systemically, other AOs including liver injury (AO344) and kidney failure (AO759) were added as a consequence of hyperinflammation or thrombosis (Vinken, 2021; Wittwehr et al., 2021).

## 4 Discussion

With the CIAO project, for the first time, a viral disease was structured within AOPs. Conceptually, a network graph can quickly capture information and an appropriate layout can be instrumental in understanding complex interdependent data (Newman, 2003). Visualization tools used in this study provided semi-automatic processes to import AOP-Wiki information and construct a primary network, highlighting inconsistencies. The final AOP network developed in this study enables visualization of the interrelations between the many perturbed biological events along the four phases of the disease and via the different entry routes of the virus for better understanding of the complex disease. However, generating a COVID-19 relevant AOP network underlined challenges in application of the framework to multi-organ complex pathogenesis involving many routes of exposure.

### 4.1 Challenges and recommendations to derive biologically relevant AOP networks

#### 4.1.1 Network quality depends on the information in the Wiki

A necessary caution to consider when interpreting AOP networks is that derivation of networks is limited by the scope of knowledge captured in the AOP-Wiki. The nodes and connections reflected in an AOP network are only those for which AOP descriptions have been developed (Villeneuve et al., 2018).

Besides, gaps in the individual AOPs, unequal quality of the KEs description or of the evidence provided for KERs still represent an important limitation in building biologically relevant AOP networks. There is no explicit quality assurance for AOPs in the AOP-Wiki, except when they have gone through a review process according to the OECD guidance (OECD, 2018). AOPs on the OECD work plan benefit from coaching that guide the authors in developing AOP consistent with the framework guidance (OECD, 2018) making those AOPs compliant for future scientific review. The process ensures the development of high quality AOPs fit for use in the research and regulatory science context.

In the present study, the developed AOPs have not yet completed the OECD process, hence they were unequal in their quality and completeness. In general, field information inside KE pages was more complete than in KER pages. A workshop was organized to guide CIAO participants in their efforts to allocate COVID-19 related evidence to KERs while KEs have to contain general and methodology information. The issue of missing or incomplete terms was a major point of focus for the CIAO Ontology Group. In the AOP-Wiki, the authors are required to fill-in several fields/entries, and inevitably, a great majority of those are left empty or filled-in by generic (e.g., “ Cell” or “ Organ”) terms. Therefore, a bug tracking system, such as the one described below, would allow for warning both the AOP authors and developers against such issues when identified. A concern often raised by AOP authors is that they are not always able to identify a specific term in the current ontology set in the AOP-Wiki. In such cases, the selected ontology needs to be amended with the required information. A suitable alternative for the AOP-Wiki community is to reach out to ontology providers (e.g., the UBERON experts) that are open to recommendations, in order to dictate new terms or required fixes in the respective ontologies.

#### 4.1.2 Importance of ontology harmonization

This study has underlined the importance of terminology harmonization in AOP-Wiki for network building. For example, AO1276 *Lung fibrosis* and AO1458 *Pulmonary fibrosis* did not converge as one node in the computationally generated network. This necessitated renaming of the AO title manually to enable connectivity in the network (Supplementary Material S1). Similarly, additional KE titles required manual harmonization before their inclusion in the network. This issue is not specific to COVID-19 and has been encountered in the AOP community since its onset (Ives et al., 2017). The importance of using standardized terminologies to describe the main concepts in an AOP and to avoid fragmentation in the way terms are described, will enable AOPs to be more interoperable with other domains, such as the regulatory world or PubMed. Most importantly, standardization and harmonization of terms will enable the communication in machine-readable format, important for downstream applications, such as reasoning and network visualization.

As discussed previously, the initial efforts focused on automatically identifying consolidation opportunities at the KE title level. However, owing to the lack of KE title standardization, this effort had to be mostly conducted manually. While this is not an easy-to-tackle problem, the CIAO Ontology group assessed the feasibility of adopting the terms from Medical Dictionary for Regulatory Activities (MedDRA), as a first step towards standardization and increasing the interoperability with the regulatory world. MedDRA consists of standardized medical terminology, developed since late 1990s by the International Council for Harmonization of Technical Requirements for Pharmaceuticals for Human Use (ICH), to facilitate sharing of regulatory information internationally for medical products used by humans. MedDRA is also the standard terminology for Adverse Event reporting in the FDA’s Adverse Event Reporting System (FAERS), as well as in other pharmacovigilance databases (e.g., Vigibase). For these reasons, it was considered as a suitable terminology for AO titles as it can better describe stressor-induced AOs observed in clinical settings. The CIAO Ontology Group’s attempt to map AO titles to MedDRA overall resulted in good coverage, as the majority of terms were successfully mapped to MedDRA Preferred Terms, e.g., KE1841 (Encephalitis). In other cases, AO titles were mapped to the most closely related MedDRA term, e.g., KE1875 (Cerebrovascular disease (stroke) was mapped to MedDRA PT Cerebrovascular accident.

Towards a more automated way to tackle such inconsistencies, an additional recommendation was made for KE titles to be automatically generated and composed of terms that exist in the KE components table, a field which, by design, allows for ontology-based annotations of KEs. This is the case of the KE components’ Process and Object fields, both of which rely on a set of ontologies, such as Gene Ontology (GO). The end goal would be to have a structured KE title recommendation as we have for AOPs (MIE leads to AO).

During the adaptation of Biovista Vizit for the AOP-Wiki data, an automatic mapper for multiple ontologies was used as part of the import process. The results for KEs were not as successful as the manual curation since only one pair of CIAO-related KEs were identified as potential duplicates across the whole AOP-Wiki. The reason for this is that the KE titles are not structured and thus the chance to correctly map them to a controlled vocabulary is small. However, the application of the same procedure to the rest of the AOP elements (Stressors, Biological Objects, Biological Process, Cells, Organs), was a much more encouraging process since it identified a number of duplications, invalid entries and other inconsistencies in the AOP-wiki terminology.

The quality issues underlined the need for a tighter implementation of the FAIR principles, for improving findability, accessibility, interoperability and reusability of the AOPs (Wittwehr et al., 2024). First, we propose a continuous integration (CI) system based on the ontology mapper approach, that will test the current state of the AOP-Wiki or any newly submitted entry for known common issues and notify the AOP author or even refuse the entry. The full output of the CI could be published in a specific location inside the AOP-Wiki and possibly, by AOP, KE or even by author, so the users and the developers are aware of the common quality issues that have been automatically detected.

A second recommendation could be the development of a bug tracking system (BT), similar to “GitHub Issues”, that will help users and developers report quality issues, keep track of them and their dependencies and finally resolve them.

However, having in place mechanisms such as CI and BT for detecting, reporting, discussing and organizing quality issues, is not enough. Any such initiative should be coupled by a policy of addressing quality issues. For conflict resolution, one could envision a committee that can have a final decision on how a particular issue should be fixed or which solution should be applied. Finally, it should be possible for engaged individuals within the AOP-Wiki community (e.g., AOP-Wiki curators) to perform complex refactoring, e.g., deduplicating/merging content, so that such issues are readily resolved. Another recommendation could be to develop a systematic way to identify duplicate KEs extracted from the AOP-Wiki and apply it as a filter before providing the primary network.

Finally, one could learn that when embarking on a large-scale AOP-aligned collaboration, harmonization of ontology and controlled vocabulary is something that is best brought about as early as possible, and certainly not assumed as implicit.

#### 4.1.3 Need to account for organ specificity

No additional parameters such as taxonomic space, life stages, sex applicability were used to filter and restrict the network. By contrast the primary derived network visually highlighted the need for manual duplication of some KEs to account for organ specificity. Prominent example is KE1739 (Binding to ACE2), which occurs in lung cells and is also the MIE in sustentacular cells and enterocytes. Accounting for tissue specificity was essential for the representation of systemic or cross tissue effects in the AOP network. As such, according to the AOP principles, KEs are not specific to tissue or cell types. These principles have been mainly driven by the practical consideration of reusing KEs to prevent the duplication of effort when defining those KEs. However, if the downstream effects of a KE are dependent on the cell-type, tissue, or organ where the KE occurs, it may be more appropriate to define individual KEs more narrowly and look for means of sharing information across the separate KEs. As more emphasis is placed on defining KEs using biological ontologies, the options for information sharing across KEs are dramatically improved. The need to account for organ specificity has also driven the CIAO Ontology Group to consider and recommend the combination of all Biological context fields in one table, allowing more than one row (cell, tissue or even subcellular location) per KE, within a single KE page.

Taken together, addressing such challenges would not only allow for the sustainability and re-use of the CIAO-developed AOPs, but also pave the way for the development of novel AOPs addressing emerging health issues that could benefit from the collaborative aspect of the AOPs.

### 4.2 Challenges and recommendations specific to viral AOP networks

#### 4.2.1 Need to describe the SARS-CoV-2 specific infection steps

When modeling COVID-19 via AOPs, the stressor agnostic principle was challenged (Clerbaux et al., 2023). Depicting the biology of the virus was essential to capture the mechanisms underlying the disease onset and progression. Viral replication and viral load are well-established parameters correlated with poor prognosis of the disease (Brosseau et al., 2022) and most approved antivirals act on viral proteins specifically. Hence, stressor-specific KEs describing the replication cycle of SARS-CoV-2 were proposed to serve as a series of linked KEs, a reusable unit that can be integrated into the several AOPs that required SARS-CoV-2 replication to mediate the outcomes (Clerbaux et al., 2023). As such, only the initial three linked KEs (KE1738, KE 1901, KE 1847) are specific to SARS-CoV-2 while the downstream KEs in the different AOPs follow the stressor agnostic principle. In addition, the MIE (KE1739: Binding to ACE2) is stressor-agnostic which maintains the MIE open to inclusion of evidence from other stressors, such as SARS-Co-V or ACE2 modulators. In line, binding to ACE2 does not only lead to SARS-CoV-2 cell entry as exemplified with ACE2 dysregulation downstream event.

#### 4.2.2 Discriminating physiological *versus* pathological inflammation in AOPs

The network showed that the diverse AOs are interrelated and share common signaling pathways and associated molecules, at the core of which is the inflammatory response, playing a central role in initiating a cascade of events locally and systemically spanning over several tissues and culminating in a variety of AOs. The complexity of the disease process and conflicting information on the timing and levels of expression of several pro/anti-inflammatory mediators, makes it challenging to draw a clear picture of the immune response onset and sequential progression towards severity. Many of these events occur in parallel, creating physiological havoc, whereby host defense mechanisms assume host destruction roles. In order to tentatively capture this temporal and quantitative aspect of the immune response, the network was organized visually into the four phases of the disease: disease onset (viral replication), early cellular and tissue damage; immune and vascular response and late adverse outcomes. In addition, to discriminate between physiological and pathological inflammation, the AO hyperinflammation (KE1868) was created which defines *per se* a pathological situation. However, this might not be sufficient and a quantification aspect of the AOPs might be needed along with a combination of relevant markers.

#### 4.2.3 Utility of an AOP network for a viral disease

Responses to stressor exposure are more complex than the simple one-biological-perturbation to one-adverse-outcome portrayed in individual AOPs. AOP network can aid in understanding and in predicting effects of more realistic complex exposure scenarios. As for toxicology, such network underlined the gaps in our understanding of the pathways, which can guide future needed research. In the context of a viral disease, such a network could provide a structure to integrate multi-omics data from patients into a comprehensive understanding of the pathways at play, notably regarding the inflammatory response. The identification of potential biomarkers could also be of interest, especially for discriminating pathological inflammation. Such network can also serve to capture the influence of the modulating factors such as age, sex, pre-existing comorbidities in order to understand why some patients are more vulnerable than others (Clerbaux, Albertini, et al., 2022). However, such utilities have still to be operated. Finally, the AOP framework was inherently interdisciplinary which was needed to tackle such a complex disease and to support a collaborative large-scale crowdsourced effort (Carusi et al., 2023).

## 5 Conclusion

This study provided insight into the complex processes and interconnections between perturbed key biological events in COVID-19, as well as the reusability of the knowledge and framework from another field, e.g., toxicology. The study highlighted the challenges in developing an AOP network, such as the need to adopt globally the use of controlled vocabularies and ontologies, the need to consider organ specificity, the gaps in the individual AOPs, the quality of the current KEs and KERs and the challenges specific to applying this framework to a viral disease. The value and impact of the effort presented here stem from how the gathered knowledge about the disease is stored within AOPs in a manner that supports its reuse and uptake within other areas of research, including toxicology and any potential future pandemics caused by other types of pathogens. The current work demonstrates the potential of the AOP framework to support interdisciplinary research while lessons learnt aim to serve ongoing or future AOP-aligned initiatives.

## Supplementary Material

Supplement1

## Figures and Tables

**TABLE 1:** The list of 24 AOPs of which 17 (in bold) were considered for inclusion in the primary network.

	AOP ID	AOP description	Project	Included in the primary network
Viral AOP	**AOP430**	Binding of SARS-CoV-2 to ACE2 leads to viral infection	1.96	Yes
Inflammatory and vascular AOPs	**AOP379**	Binding of SARS-CoV-2 to ACE2 leads to thrombosis	1.96	Yes
	**AOP392**	Bradykinin and fibrinolytic dysregulation leads to hyperinflammation	1.96	Yes
	**AOP412**	Endothelial cell dysfunction leads to thrombo-inflammation	1.96	Yes
	AOP385	Binding to ACE2 leads to microvascular dysfunction	CIAO	Yes, but empty
Lung AOPs	**AOP320**	Binding of SARS-CoV-2 to ACE2 leads to acute respiratory distress (ARDS)	1.96	Yes
	AOP377	TLR9 activation leads to ARDS	CIAO	No, not following OECD guidance
	**AOP173**	Substance interaction with the lung resident cell membrane components leads to lung fibrosis	**1.32**	Yes
	**AOP319**	Inhibition of ACE2 leads to lung fibrosis	1.96	Yes
	AOP382	AT1R agonism leading to lung fibrosis	CIAO	Yes, but merged
	**AOP302**	Lung surfactant function inhibition leading to decreased lung function	**1.87**	Yes
Neuro AOP	**AOP374**	Binding of SARS-CoV-2 to ACE2 in brain cells leads to neuroinflammation resulting in encephalitis	1.96	Yes
	**AOP394**	Binding of SARS-CoV-2 to ACE2 in olfactory epithelium leads to impaired olfactory function (short term anosmia)	1.96	Yes
	**AOP395**	Binding of SARS-CoV-2 to ACE2 in pericytes leads to intravascular coagulation resulting in stroke	1.96	Yes
	AOP381	Binding to ACE2 leads to dysgeusia	CIAO	Yes, but empty
Gut AOPs	**AOP422**	Binding of SARS-CoV-2 to ACE2 in enterocytes leads to intestinal barrier disruption	1.96	Yes
	**AOP428**	Binding to ACE2 in enterocytes leads to gut dysbiosis	1.96	Yes
Liver AOP	**AOP383**	Inhibition of ACE2 leads to liver fibrosis	CIAO	Yes, but empty
		Binding to ACE2 in lungs leads to thrombosis and liver injury	CIAO	No, not in Wiki
		Hyperinflammation leads to liver injury	CIAO	No, not in Wiki
Kidney AOP		Binding to ACE2 in lungs leads to thrombosis and kidney injury	CIAO	No, not in Wiki
		Hyperinflammation leads to kidney injury	CIAO	No, not in Wiki
Heart AOP	AOP427	Downregulation of ACE2 leads to heart failure	CIAO	No, not following OECD guidance
	AOP426	Binding to ACE2 in pericytes leads to myocardial infarction	CIAO	No, not following OECD guidance

**TABLE 2:** Harmonization of KE titles of duplicated KEs.

Corresponding KE 1	Corresponding KE 2	Harmonized KE title
1458 Pulmonary fibrosis	1276 Lung fibrosis	Pulmonary fibrosis
1501 Increased, extracellular matrix deposition	68 Accumulation, Collagen	Increased, extracellular matrix deposition
1752 Increased Angiotensin II	1743 Increase plasma Ang II	Increased Angiotensin II
1869 Diminished protective oxidative stress response	1115 Increased, Reactive oxygen species	Oxidative stress response
1496 Increased, secretion of proinflammatory and profibrotic mediators	87 Release, Cytokine	Increased, secretion of proinflammatory mediators
1497 Increased, recruitment of inflammatory cells	1750 Increased inflammatory immune responses	Increased, recruitment of leukocytes
1740 ACE2 inhibition	1787 Downregulation, ACE2	ACE2 inhibition
1852 Increased Ang II type 1 receptor (AT1R)	1851 Binding of agonist, Angiotensin II receptor type 1 receptor (AT1R)	Increased Ang II type 1 receptor (AT1R)
1868 Hyperinflammation	1844 Systemic inflammatory response syndrome	Hyperinflammation

**TABLE 3:** AOP network-driven identification of new KERs.

#	Upstream KE	Downstream KE
1	1847 Increased coronavirus production	1825 Increase, Cell death
2	1825 Increase, Cell death	Increased, secretion of proinflammatory mediators (KEs 1,496, 87)
3	Secondary/internal routes of entry	1874 Blood brain barrier disruption
4	Secondary/internal routes of entry	1931 Intestinal barrier, disruption
5	1868 Hyperinflammation	759 Increased kidney failure
6	1846 Thrombosis and Disseminated intravascular coagulation	759 Increased kidney failure
7	1846 Thrombosis and Disseminated intravascular coagulation	344 Liver injury
8	1868 Hyperinflammation	344 Liver injury
9	1872 Olfactory epithelium degeneration	188 Neuroinflammation
10	1874 Blood brain barrier disruption	188 Neuroinflammation
11	1868 Hyperinflammation	1874 Blood brain barrier disruption
12	Increased, recruitment of leukocytes (KEs 1,497, 1750)	1954 Gut microbiota, alteration
13	1868 Hyperinflammation	1954 Gut microbiota, alteration
14	1931 Intestinal barrier, disruption	1932 Gastrointestinal disorders
15	1931 Intestinal barrier, disruption	Increased, recruitment of leukocytes (KEs 1,497, 1750)
16	1931 Intestinal barrier, disruption	1868 Hyperinflammation
17	1954 Gut microbiota, alteration	1931 Intestinal permeability, increased
18	1954 Gut microbiota, alteration	1932 Gastrointestinal disorders

## Data Availability

The original contributions presented in the study are included in the article/Supplementary Material, further inquiries can be directed to the corresponding authors.
